# Screening of selected ageing-related proteins that extend chronological life span in yeast *Saccharomyces cerevisiae*

**DOI:** 10.1038/s41598-021-03490-7

**Published:** 2021-12-17

**Authors:** Jee Whu Lee, Tee Gee Ong, Mohammed Razip Samian, Aik-Hong Teh, Nobumoto Watanabe, Hiroyuki Osada, Eugene Boon Beng Ong

**Affiliations:** 1 Institute for Research in Molecular Medicine (INFORMM), Universiti Sains Malaysia, 11800 USM, Penang Malaysia; 2 USM-RIKEN International Centre for Ageing Science (URICAS), Universiti Sains Malaysia, 11800 USM, Malaysia Penang; 3 School of Biological Sciences, Universiti Sains Malaysia, 11800 USM, Penang Malaysia; 4 Centre for Chemical Biology, Universiti Sains Malaysia, 11900 Bayan Lepas, Penang Malaysia; 5 Bioprobe Application Research Unit, RIKEN Centre for Sustainable Resource Science, 2-1 Hirosawa, Wako, Saitama 351-0198 Japan; 6 Chemical Biology Research Group, RIKEN Centre for Sustainable Resource Science, 2-1 Hirosawa, Wako, Saitama 351-0198 Japan

**Keywords:** Biochemistry, Proteins

## Abstract

Ageing-related proteins play various roles such as regulating cellular ageing, countering oxidative stress, and modulating signal transduction pathways amongst many others. Hundreds of ageing-related proteins have been identified, however the functions of most of these ageing-related proteins are not known. Here, we report the identification of proteins that extended yeast chronological life span (CLS) from a screen of ageing-related proteins. Three of the CLS-extending proteins, Ptc4, Zwf1, and Sme1, contributed to an overall higher survival percentage and shorter doubling time of yeast growth compared to the control. The CLS-extending proteins contributed to thermal and oxidative stress responses differently, suggesting different mechanisms of actions. The overexpression of Ptc4 or Zwf1 also promoted rapid cell proliferation during yeast growth, suggesting their involvement in cell division or growth pathways.

## Introduction

Ageing is a process that occurs at the cellular to the organismal level. Over the years, various model organisms have been used to study various aspects of ageing and the yeast, *Saccharomyces cerevisiae*, has stood out as a suitable model for cellular ageing studies. In yeast, chronological life span (CLS) is defined as the length of time that a cell is viable in a nondividing quiescent state and can re-enter the cell cycle when stimulated^1–3^. The yeast CLS assay was developed to model ageing of nondividing cells of higher organisms^3,4^ and has been used to study the molecular changes in postmitotic cells such as neurons and muscle cells^5,6^.

The CLS assay has been used to identify ageing-related genes. For example, a CLS screen of deletion mutants revealed genes that significantly extended CLS (*ACB1*, *CKA2*, *TRM9*) and the deletion of these genes promoted heat-shock tolerance^7^. Another screen of deletion mutants found that while autophagy defective mutants (*ATG* mutants) decreased CLS, highly conserved de novo purine biosynthesis pathway defective mutants (the *ADE* mutants) extended CLS to the same degree as calorie restriction^8^. In another CLS screen, *CYR*1 and *SCH9* mutants were found to extend CLS and increase thermal and oxidative stress tolerances^9^. In contrast, CLS regulation can also be investigated by overexpressing proteins in *S. cerevisiae*. As an example, overexpression of both Sod1 and Sod2 was shown to extend CLS of *S. cerevisiae* by 30%, however the overexpression of either Sod1 or Sod2 alone could only lead to minor CLS extension in budding yeast^10^. In addition, overexpression of Sir2 also extended CLS in *S. cerevisiae*^11^*.*

Ageing research in *S. cerevisiae* has contributed to the discovery of various genes associated with longevity and major longevity signalling pathways such as the sirtuin pathway and target of rapamycin (TOR) pathway^5,12^. Life span extension by the overexpression of Sir2 can inhibit the TOR pathway which is also highly conserved in other divergent eukaryotes (worms, flies and mice)^13–22^. The downregulation of two nutrient signalling pathways, target of rapamycin (TOR) and cyclic adenosine monophosphate dependent protein kinase A (cAMP-PKA), was reported to extend longevity by activating Rim15, a downstream target converged by the two pathways^23–25^. Activated Rim15 then induces the activation of the stress-resistance transcription factors Gis1 and Msn2/4 that promote cellular resistance towards stresses such as osmotic stress, thermal stress, oxidative stress and nutrient starvation, leading to CLS extension^2,9,10,23–26^. Such findings show that the increase of stress tolerance factors contributes to CLS extension.

A yeast CLS assay is initiated by growing yeast cells in synthetic complete (SC) medium for 24–48 h until they reach stationary phase^9^. Colony forming unit (CFU) count is generally used to determine survival of cells during CLS. This method is performed by doing serial dilution and plating the diluted cultures on nutrient-rich agar. The number of colonies are then counted to calculate cell viability. However, the CFU method is laborious, time-consuming, and is unpractical for screening of many genes simultaneously^13,27^. Therefore, alternative methods for measuring CLS in a rapid manner such as outgrowth kinetics assay^1^, barcoded competition-based assay^8^ and fluorescent cell counting^28^ were developed.

Here, we performed an overexpression screen of 15 ageing-related proteins in *S. cerevisiae* using a 96-well plate based CLS assay. We found that the overexpression of Ptc4, Zwf1, Sme1, Cpr3, Kss1 or Sod1 could extend yeast CLS. We then characterised the top three CLS-extending proteins (Ptc4, Zwf1, Sme1) in terms of their stabilities and stress responses during chronological ageing, and the proteins’ effects on cell proliferation. We found Ptc4, Sme1 and Sod1 were stable, while Zwf1 appeared degraded after eight days of ageing. Next, we found that Ptc4 contributed to thermal and oxidative stress tolerances. Zwf1, Sme1 and Sod1 did not show stress tolerance in the stress assays after prolonged ageing. Finally, Ptc4 or Zwf1 overexpression promoted rapid cell proliferation during yeast growth.

## Methods

### Strain, growth condition and transformation

The *S. cerevisiae* strains used in this study were MLC30M (*MATa trp1-1 leu2-3,112 his3-11,15 ura3-1 ade2-1 can1-100 yrs1::HIS3 yrr1::TRP1 pdr1::hisG pdr3::hisG*) (NBRP, Japan)^29,30^ and BY4741 (*MATa leu2Δ0 ura3Δ0 his3-Δ1 met15Δ0*) (NBRP, Japan)^31,32^. The gene deletion mutants of BY4741 were purchased from Euroscarf, Germany. The deleted genes were replaced by *kanMX4*. Yeast cells were cultured at 30 °C in Yeast Peptone Dextrose (YPD) medium (10 g/L yeast extract, 20 g/L peptone, and 20 g/L dextrose) (HIMEDIA, India) for general growth and CLS screen of the mutants. Synthetic complete (SC) medium consisting of 2% glucose (HIMEDIA, India), 0.67% yeast nitrogen base with ammonium sulphate (HIMEDIA, India), and amino acids (HIMEDIA, India) at appropriate concentrations and lacked certain amino acids (uracil, leucine) were used for plasmid selection and protein expression assays. The concentrations of amino acids in SC medium are listed in Table 1. Yeast transformation was performed as described^33^, and transformants were selected from synthetic complete minus uracil (SC-Ura) agar.

**Table 1:** Final concentrations of amino acids in SC medium.

Amino acid	Final concentration in SC medium (mg/L)
L-Isoleucine	30
L-Valine	150
Adenine sulphate	20
L-Arginine	20
L-Lysine	30
L-Methionine	20
L-Phenylalanine	50
L-Threonine	200
L-Tyrosine	30
L-Histidine	24
L-Tryptophan	24
L-Leucine	36

### Selection and cloning of ageing-related genes

A total of 97 human age-related genes were selected from National Center for Biotechnology Information (NCBI) Gene database (https://www.ncbi.nlm.nih.gov/gene, accessed December 2017) by using the keywords “lifespan” and “*Homo sapiens*” to filter the selection. After cross-referencing with *Saccharomyces* Genome Database (SGD), seven yeast age-related genes which are homologous to human genes were selected out of the 97 genes and among the seven genes, three genes (*POL30*, *ZWF1*, *CPR3*) have unknown effects on yeast CLS.

Literature review revealed an additional 190 ageing-related genes using the keywords “longevity”, “lifespan” or “CLS” in the PubMed database (https://pubmed.ncbi.nlm.nih.gov/, accessed December 2017). Out of the 190 genes, 111 genes have unknown effects on yeast CLS based on their status in SGD. Ultimately, nine genes (*PTC4*, *SME1*, *KSS1*, *RPN11*, *PAP1*, *HSC82*, *UBP13*, *CDC6*, *ESP1*) were chosen based on their size, subcellular location, protein function, and gene ontology. Three other genes (*SOD1*, *AIM14*, *UMP1*) with known effects on CLS were selected as controls. Altogether, 15 genes were selected for screening (Supplementary Table S1).

The pYEX-BX [AmpR, ori(PUC), CUP1 promoter, leu2-d, URA3, ori(2μ), multiple cloning site (MCS) of *Bam*HI, *Sal*I, *Pst*I, *Eco*RI] (TaKaRa, Japan) had been modified to generate the cloning plasmid pYEX [Amp^R^ ori(pUC) CUP1 leu2-d URA3 ori(2μ) MCS (*Sal*I, *Pst*I, *Sac*II, *Xho*I, *Nhe*I, *Sac*I, *Xma*I/*Sma*I, *Bam*HI) FLAG-tag 6 × His-tag]^34^.The selected genes were then cloned into the cloning plasmid pYEX using standard molecular biology protocols. Briefly, the genes were amplified by PCR from the genome of S288C (*MATα SUC2 mal mel gal2 CUP1 flo1 flo8-1 SSD1-v1*) (NBRP, Japan), purified and inserted recombinantly by homologous cloning into linearised cloning plasmid pYEX, in *Escherichia coli* DH5α^35^. The reaction mixture required for cloning contained ~ 1000 ng cloning plasmid pYEX linearised by restriction enzymes *Xho*I and *Xma*I, ~ 600 ng amplified gene, 1 × KCM buffer and distilled water. The reaction mixture was mixed with 30 μL DH5α competent cells and cooled at 4 °C for 1 h, then heat-shocked at 42 °C for 90 s, and lastly cooled at 4 °C for 5 min. An amount of 50 μL Super Optimal broth with Catabolite repression (SOC) was added into the mixture. The mixture was incubated for 4 h and spread on Luria–Bertani (LB) agar with 0.1 mg/mL ampicillin for transformant selection. The transformant colonies were detected by colony PCR and the clones were then verified by DNA sequencing (Apical Scientific, Malaysia).

### Generation of control plasmid

The cloning plasmid pYEX was modified to generate a control plasmid pYEX used in this study. The cloning plasmid pYEX was linearised by restriction enzymes *Xho*I and *Xma*I and purified. Two single-stranded complementary oligonucleotides, Oligo_pYEX_Forward (5’-GGTGGTTCTGGTGGCGGCTCTGGCCCGCGGATGCCTCCTCCATACCAGCCTCTCGGAGGA-3’) and Oligo_pYEX_Reverse (5’-TCCTCCGAGAGGCTGGTATGGAGGAGGCATCCGCGGGCCAGAGCCGCCACCAGAACCACC-3’), at concentration of 45 μM each, were annealed in 1 × T4 ligase buffer using thermal cycler chamber (Bio-Rad MyCycler™, United States). The thermal profile was set in the order of the following: dissolution at 95 °C for 10 min, annealing at 25 °C for 12 min, temperature drop by 0.1 °C/s and 25 °C for infinity. The reaction mixture which contained the purified linearised cloning plasmid pYEX, 45 μM double-stranded oligonucleotides, 1 × KCM buffer and distilled water, was prepared. The volume (μL) ratio of cloning plasmid pYEX to oligonucleotides, 2:15 was used. The reaction mixture was mixed with 30 μL DH5α competent cells and cooled at 4 °C for 1 h, then heat-shocked at 42 °C for 90 s, and lastly cooled at 4 °C for 5 min. An amount of 50 μL LB broth was added into the mixture. The mixture was incubated for 4 h and spread on LB agar with 0.1 mg/mL ampicillin for transformant selection. The transformant colonies were grown overnight in LB broth containing 0.1 mg/mL ampicillin and the plasmids extracted from the clones were then verified by DNA sequencing (Apical Scientific, Malaysia). The DNA sequences of cloning plasmid pYEX and control plasmid pYEX were listed (Supplementary Table S2).

### Chronological life span assay

The yeast strain MLC30M was first transformed with recombinant plasmid pYEX carrying the ageing-related genes or control plasmid pYEX to prepare aged cultures. A single colony was selected and grown overnight in a bijou bottle with 1 mL of SC-Ura medium at 30 °C with shaking at 200 rpm. A total of three colonies was selected as three biological replicates of protein overexpression strain or control plasmid pYEX strain. An amount of 50 μL of each overnight culture was inoculated into 5 mL of fresh synthetic complete minus uracil minus leucine (SC-Ura-Leu) medium with 500 μM CuSO_4_ in a universal bottle. The cultures in universal bottles were incubated at 30 °C with shaking at 200 rpm for eight days. The day after an initial 48 h of growth (after two days of incubation in universal bottle) is defined as day 0 of stationary phase. On day 0, 2, 4, and 6 of stationary phase, an aliquot of 2 μL of aged yeast culture from each universal bottle was transferred into a well of 96-well flat bottom microplate (IWAKI) containing 98 μL SC-Ura-Leu medium with 500 μM CuSO_4_. The protocol for aged culture preparation, aged culture transfer to microplate for outgrowth absorbance measurement by microplate reader, and the formulae of calculating survival percentage and doubling time were adapted from Murakami et al.^1^.

In the primary screen, the yeast cultures in the 96-well microplate were incubated at 30 °C with continuous shaking at 210 rpm for 24 h in Bio Microplate Reader HiTS (Cosmo Bio, Japan). Absorbances at a wavelength of 600 nm (A_600_) of the cultures in the 96-well microplate were measured by the reader every 30 min until 24 h. From the primary screen, the protein overexpression strains with higher relative absorbances (average absorbance value of each strain at 24 h divided by average absorbance value of the control strain at 24 h) than the control strain on day 6 were selected for a confirmatory screen. In the confirmatory screen, the cultures in a 96-well microplate were incubated at 30 °C with continuous shaking at 210 rpm up to 48 h in the reader. Absorbances (A_600_) were measured every 30 min until 48 h. Survival percentage of each replicate of the protein expression strain and control strain was determined by the formula: S_n_ = $\frac{1}{{2}^{(\frac{\Delta {t}_{n}}{{\delta }_{r}})}} \times 100\%$, whereby S_n_ is survival on day n, ∆t_n_ is time shift from day 0 to day n at A_600_ 0.2, calculated from the linear regression equation of natural logarithm of A_600_ and δ_r_ is the doubling time of each replicate. This δ_r_ was determined from the maximal slope of the semilog plot of absorbance against time and was defined as the average of at least three δ values for every consecutive pair of absorbance values for that replicate. Doubling times (δ) were calculated between every consecutive pair of absorbance values, by the formula: δ = $\frac{\text{ln}(2)}{(\frac{\text{ln}\left({\text{A}}_{2}\right)-\text{ln}\left({\text{A}}_{1}\right)}{{\text{t}}_{2} - {\text{t}}_{1}})}$, whereby A_1_ and A_2_ are successive absorbance values while t_1_ and t_2_ are the times between measurement of successive absorbance. Survival percentage of each replicate of each strain was then averaged^1,36^.

In CFU assay, the aged cultures of MLC30M were prepared in universal bottles as described in the outgrowth kinetics assay, except that each overnight culture was standardised to A_600_ of ~ 0.2 (1.7–2.5 × 10^7^ cells/mL) in SC-Ura-Leu with 500 μM CuSO_4._ Three biological replicates of each aged sample were prepared. A volume of 400 μL of the standardised culture was added into a universal bottle containing SC-Ura-Leu with 500 μM CuSO_4_ to a final volume of 5 mL with 1–2 × 10^6^ cells/mL. The cultures in universal bottles were incubated at 30 °C with shaking at 200 rpm for eight days. On day 0, 2, 4, and 6 of stationary phase, 150 μL of each culture was centrifuged at 5000 rpm, for 2 min and the supernatants were discarded. The pellet of each culture was resuspended in distilled water to standardise the suspension to A_600_ of ~ 0.4 (3.4–4.8 × 10^7^ cells/mL). The standardised cultures were diluted in distilled water to make tenfold serial dilution and spread on SC-Ura-Leu agar with 500 μM of CuSO_4_. The agar plates were then incubated for five days at 30 °C for colony counting. The CFU assay was repeated on day 2, 4 and 6. Survival percentage of each replicate of the protein expression strains and control strain was determined by the formula: S_n_ = $\frac{\text{replicate's CFU}/\text{mL on day n }}{\text{replicate's CFU}/\text{mL on day }0 } \times 100 \%$, whereby S_n_ is survival on day n.

In the CLS screen of gene deletion mutants, the *ptc4*∆, *zwf1*∆, *sme1*∆, *sod1*∆ mutants and control wild-type BY4741 were first streaked on YPD agar and incubated for two days. A single colony was selected and grown overnight in a bijou bottle with one millilitre of YPD medium at 30 °C with shaking at 200 rpm. Three colonies were selected as three biological replicates of each sample strain. Each overnight culture was standardised to A_600_ of ~ 0.3 in YPD medium. A volume of 400 μL of the standardised culture was added into a universal bottle containing YPD medium to a final volume of 5 mL. The cultures in universal bottles were incubated at 30 °C with shaking at 200 rpm for 28 days. On day 0, 2, 4, 6, 12, 16, 26 of stationary phase, 150 μL of each culture was centrifuged at 5000 rpm for 2 min and the supernatants were discarded. The pellet of each culture was resuspended in YPD medium to standardise the suspension to A_600_ of ~ 0.3. An aliquot of eight microlitres of standardised ageing yeast culture was transferred into a well of 96-well flat bottom microplate (IWAKI) containing 92 μL YPD medium for outgrowth^1^. The yeast cultures in the 96-well microplate were incubated at 30 °C with continuous shaking at 210 rpm for 24 h in Bio Microplate Reader HiTS (Cosmo Bio, Japan). Absorbances (A_600_) of the cultures in the 96-well microplate were measured by the reader every 30 min until 24 h.

### Western blotting

The aged cultures of MLC30M were prepared in universal bottles as described in the CFU assay. Every two days (day 0, 2, 4 and 6), an amount of 750 μL of yeast cells in each universal bottle was harvested. The cells were standardised by suspending the cell pellet in sterile water to A_600_ of ~ 0.4. An amount of 800 μL of standardised cells was incubated in 0.4 M NaOH for 7 min at room temperature and was centrifuged at 5000 rpm for 2 min. The supernatants were discarded. Each cell pellet was resuspended in 50 μL of 2 × SDS buffer and boiled at 95 °C for 10 min. The cells were centrifuged and 10 μL of each supernatant containing proteins was separated on 12% SDS-polyacrylamide gels in a BIO-RAD Mini-PROTEAN Tetra Cell at 100 mAmp for running four gels simultaneously. BIO-RAD Trans-blot SD Semi-dry Transfer Cell was used to transfer proteins from the gels to nitrocellulose membrane at 0.32 A (for four gels) for 2 h. The primary and secondary antibodies used for detection of the overexpressed proteins were 6 × His-tag monoclonal antibody (Thermo Fisher Scientific, USA) diluted at 1:1,200, and goat anti-mouse IgG(H + L) horseradish peroxidase (HRP)-conjugated antibody (Thermo Fisher Scientific, USA) diluted at 1:1,000 respectively.

For checking protein expression level during growth, a single colony transformed with recombinant pYEX carrying *PTC4* or pYEX only (as a control) was selected and grown overnight in a bijou bottle with 1 mL of SC-Ura medium at 30 °C with shaking at 200 rpm. Each overnight culture was standardised to A_600_ of ~ 0.2 (1.7–2.5 × 10^7^ cells/mL) in SC-Ura_._ A volume of 400 μL of the standardised culture was added into a universal bottle containing SC-Ura to a final volume of 5 mL with 1–2 × 10^6^ cells/mL. The cultures in universal bottles were incubated at 30 °C with shaking at 200 rpm for 22–23 h. The overnight cultures were standardised to A_600_ of ~ 0.2 (1.7–2.5 × 10^7^ cells/mL) in SC-Ura-Leu with different concentration of CuSO_4_ (0 μM, 4 μM, 8 μM, 15 μM, 20 μM, 25 μM, 50 μM, 100 μM, 200 μM, 300 μM, 400 μM, 500 μM). A volume of 400 μL of the standardised culture was added into a universal bottle containing SC-Ura-Leu with different concentration of CuSO_4_ to a final volume of 5 mL with 1–2 × 10^6^ cells/mL. The cultures in universal bottles were incubated at 30 °C with shaking at 200 rpm until reaching A_600_ ~ 0.2 (mid-log phase). An amount of 1,400 μL of yeast cells in each universal bottle was harvested. The cells were standardised by suspending the cell pellet in sterile water to A_600_ of ~ 0.4. An amount of 700 μL of standardised cells was incubated in 0.4 M NaOH and prepared for transblotting as described earlier in western blotting of ageing-related proteins. The primary and secondary antibodies used for detection of the overexpressed proteins were 6 × His-tag monoclonal antibody (Thermo Fisher Scientific, USA) diluted at 1:1500 and goat anti-mouse IgG(H + L) HRP-conjugated antibody (Thermo Fisher Scientific, USA) diluted at 1:1000 respectively. The primary and secondary antibodies used for detection of the loading control α-tubulins were anti-α-tubulin antibody (Abcam, UK) diluted at 1:1200 and goat anti-rabbit IgG(H + L) HRP-conjugated antibody (Abcam, UK) diluted at 1:1500 respectively.

### Stress assay

The aged cultures of MLC30M were prepared in universal bottles as described in the CFU assay. Each sample (protein overexpression strain or control plasmid pYEX strain) was prepared with three biological replicates. On day 6 of stationary phase, an amount of 800 μL of cells was standardised by centrifuging at 5000 rpm for 2 min and suspending the cell pellet in sterile water to A_600_ of ~ 0.4 (3.4–4.8 × 10^7^ cells/mL). An amount of 300 μL of standardised culture was exposed to thermal stress for 30 min at 55 °C, while another 300 μL of standardised culture was exposed to oxidative stress by being treated with 2 mM hydrogen peroxide (H_2_O_2_) for 30 min at 30 °C. The other 300 μL of standardised culture was not treated with stress. The nonstress- and stress-treated cultures were diluted in distilled water to perform tenfold serial dilution (10^–2^, 10^–3^, 10^–4^, 10^–5^). The tenfold serially diluted nonstress- and thermal stress-treated cultures were spread on SC-Ura-Leu agar with 500 μM of CuSO_4_ while the tenfold serially diluted oxidative stress-treated cultures were spread on SC-Ura-Leu agar with 500 μM of CuSO_4_ and 2 mM H_2_O_2_. The agar plates were incubated for 5 days at 30 °C. Colonies of the strains formed after 5 days were counted. Survival percentage (S) of each replicate of the protein expression strains and control strain was determined by the formula: S = $\frac{\text{stress}-\text{treated replicate's CFU}/\text{mL}}{\text{nonstress}-\text{treated replicate's CFU}/\text{mL }} \times 100 \%$.

### Growth assay

A single transformed colony was selected and grown overnight in a bijou bottle containing 1 mL of SC-Ura medium at 30 °C with agitation at 200 rpm. Two colonies were selected as two biological replicates for each sample (protein overexpression strain or control plasmid pYEX strain). The overnight cultures were centrifuged at 5000 rpm for 2 min and the supernatants were discarded. The pellet of each culture was resuspended in SC-Ura-Leu to standardise the suspension to A_600_ of ~ 0.2 (1.7–2.5 × 10^7^ cells/mL). An amount of 8 μL of the suspension was mixed with 92 μL of SC-Ura-Leu at final concentration of CuSO_4_ (0 μM, 4 μM, 8 μM, 15 μM, 20 μM, 25 μM, 50 μM, 100 μM, 200 μM, 300 μM, 400 μM, 500 μM) in a 96-well flat bottom microplate (IWAKI) to reach 1–2 × 10^6^ cells/mL. Absorbances (A_600_) of the cultures were measured every 30 min until 24 h. The 96-well plate was incubated at 30 °C with continuous shaking at 210 rpm. The doubling time of each replicate was the average of at least three doubling time values for every consecutive pair of absorbance values.

### Cell count

A single transformed colony was selected and grown overnight in a bijou bottle containing 1 mL of SC-Ura medium at 30 °C with shaking at 200 rpm. Two colonies were selected as two biological replicates for each sample. The overnight cultures were centrifuged at 5,000 rpm for 2 min and the supernatants were discarded. The pellet of each culture was resuspended in SC-Ura-Leu with 100 μM CuSO_4_ to standardise the suspension to A_600_ of ~ 0.2 (1.7–2.5 × 10^7^ cells/mL). A volume of 400 μL of the suspension was added into a universal bottle containing SC-Ura-Leu with 100 μM CuSO_4_ to a final volume of 5 mL with 1–2 × 10^6^ cells/mL. The cultures in universal bottles were incubated at 30 °C with agitation at 200 rpm until the cultures reached A_600_ of ~ 0.2 (mid-log phase). A volume of 880 μL of cell culture was sonicated at 40% amplitude for 2 min to disperse the cells by using ultrasonic homogenizer LABSONIC P (Sartorius Stedim Biotech, France). Then, the dispersed cells of 10 μL were counted on a Neubauer-improved dark-lined haemacytometer (*MA*RIENFELD). Duplicate counting was performed for each replicate.

## Results

### Ptc4, Zwf1, Sme1, Cpr3, Kss1, Sod1 and Ump1 extend chronological life span in yeast upon protein overexpression

**Figure 1:**
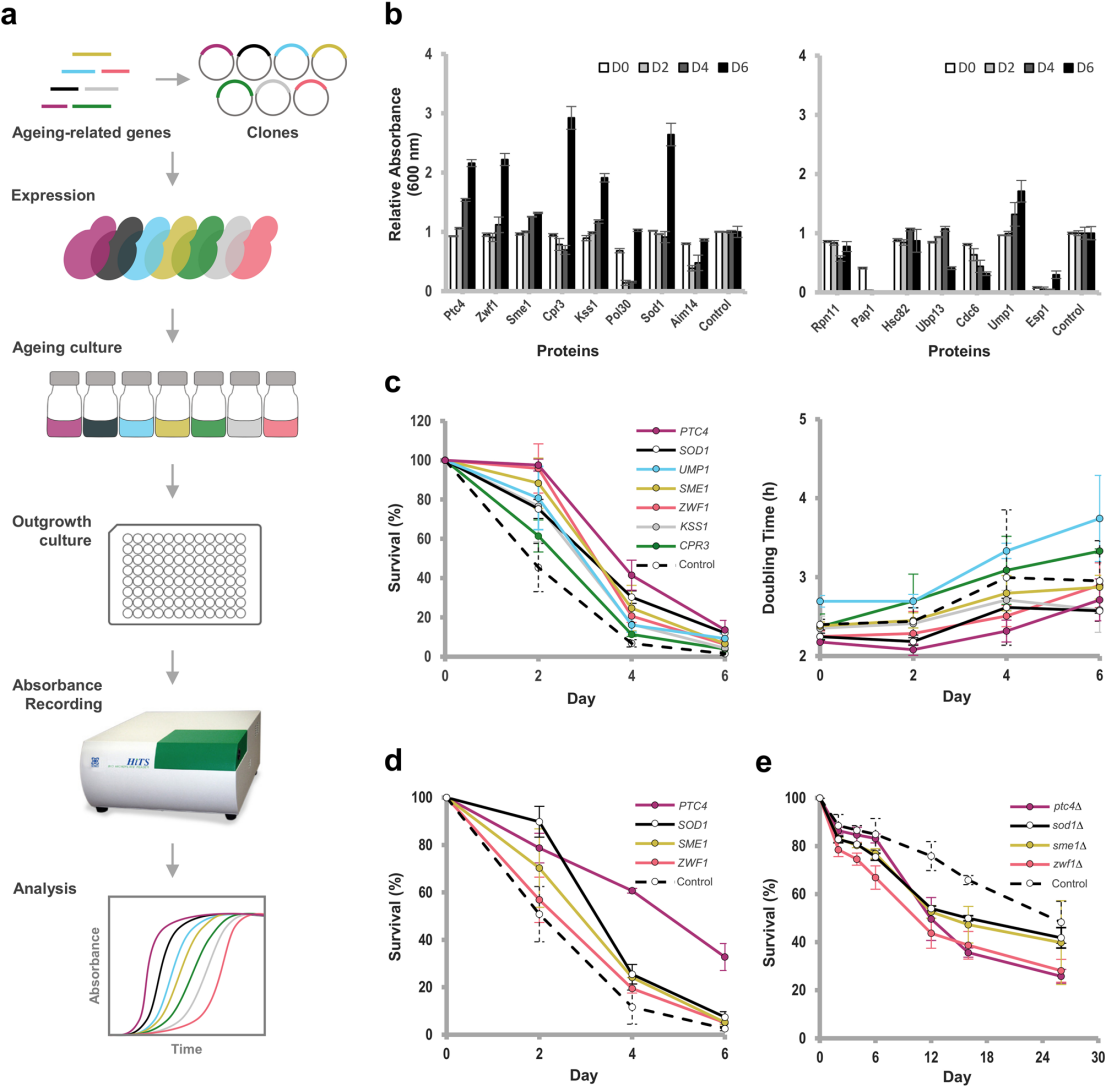
Screening for ageing-related proteins that extend chronological life span (CLS). (**a**) Overview of the screening for ageing-related proteins in CLS assay. Ageing-related genes were cloned into pYEX and overexpressed in yeast strain MLC30M. The cultures were aged in SC-Ura-Leu media with 500 μM of CuSO_4_. An aliquot of the cultures was taken every two days of incubation for outgrowth in 96-well microplate. Absorbances (A_600_) of their outgrowths were recorded on day 0, 2, 4, and 6 using Bio Microplate Reader HiTS. Growth curves were developed by plotting absorbance against time of outgrowth. (**b**) Relative absorbances of 15 protein overexpression strains compared to the control plasmid strain. Relative absorbances were calculated by dividing the strains’ average outgrowth absorbance readings at 24 h to that of the control plasmid strain (Supplementary Fig. S1). Error bars represent the standard deviations of three biological replicates. (**c**) Survival percentages and doubling times of strains that extend CLS in the confirmatory screen. The survival and doubling time values were derived from Supplementary Fig. S2. Error bars represent the standard deviations of three biological replicates. (**d**) Survival percentages of top three strains that extend CLS in CFU assay. Colonies formed by the standardised cultures at A_600_ 0.4 on day 0, 2, 4, and 6 were counted. Error bars represent the standard deviations of three biological replicates. (**e**) Survival percentages of gene deletion mutants that decrease CLS in outgrowth kinetics assay. The survival values were derived from Supplementary Fig. S3. Error bars represent the standard deviations of three biological replicates.

To determine the effects of the overexpressed proteins on yeast CLS, we overexpressed ageing-related proteins in yeast and screened them using outgrowth kinetics assay in a high-throughput manner (Fig. 1a)^1^. The yeast cultures were incubated for 8 days. For outgrowth measurement, an aliquot of culture was taken every 2 days for outgrowth in a 96-well microplate containing fresh SC-Ura-Leu media with 500 μM of CuSO_4_. Absorbances (A_600_) were measured every 30 min until 48 h using Bio Microplate Reader HiTS on each day of outgrowth (Fig. 1b, 1c). For CFU measurement, an aliquot of culture was standardised and spread on SC-Ura-Leu agar with 500 μM of CuSO_4_ in ten-fold serial dilution every 2 days. The agar plates were then incubated for colony counting (Fig. 1d). For the gene deletion mutants, an aliquot of culture was sampled for outgrowth in a 96-well microplate containing fresh YPD media. Absorbances (A_600_) were measured every 30 min until 24 h using Bio Microplate Reader HiTS on each day of outgrowth (Fig. 1e).

A selection of 15 proteins was overexpressed in the primary CLS screening assay. Twelve human homologs (Ptc4, Zwf1, Sme1, Cpr3, Kss1, Pol30, Rpn11, Hsc82 encoded Hsp90, Cdc6, Esp1, Pap1 and Ubp13) have not been investigated for their effects on yeast CLS. Three other proteins (Sod1, Aim14, Ump1) of which their effects on CLS are known were selected as overexpression controls. In the primary screen, the relative absorbances of seven overexpression strains (Ptc4, Zwf1, Sme1, Cpr3, Kss1, Sod1, Ump1) are higher than the strain carrying the control plasmid pYEX on day 6 (Fig. 1b). In accordance with previous reports, overexpression of control protein Sod1^10^ or Ump1^37^ increased CLS, while overexpression of Aim14 decreased CLS^38^ in the primary screen. The extensions of yeast CLS by the seven overexpressed proteins were verified in the confirmatory screen (Fig. 1c) which included the calculations of their survival percentages and doubling times. The overexpression of protein Ptc4, Zwf1, Sme1, Kss1 or Sod1 respectively decreased the doubling time of yeast compared to the control plasmid strain expressing a short peptide with C-terminal 6 × His-tag. The extensions of yeast CLS by the top three overexpressed proteins (Ptc4, Zwf1, Sme1) and Sod1 were reverified in CFU assay (Fig. 1d). Overall, our overexpression screen identified five proteins (Ptc4, Zwf1, Sme1, Cpr3, Kss1) that were able to extend CLS. The survivals of the strains on day 2, 4, and 6, were observed to be 2.1, 6.1, and 9.0 (Ptc4); 2.1, 3.1, and 4.2 (Zwf1); 1.9, 3.6, and 4.4 (Sme1); 1.4, 1.7, and 2.6 (Cpr3); 1.7, 2.4, and 2.8 (Kss1), and 1.7, 4.5, and 8.0 (Sod1), -times of the control plasmid strain survival respectively. The three proteins that contributed to the highest CLS extensions in yeast when being overexpressed were Ptc4, Zwf1 and Sme1. The survivals of the top three protein overexpression strains and Sod1 overexpression strain that were further tested in CFU assay, on day 2, 4, and 6 were 1.5, 5.2, and 13.0 (Ptc4), 1.1, 1.7, and 2.0 (Zwf1), 1.4, 2.1, and 2.1 (Sme1), and 1.8, 2.2, and 2.9 (Sod1), -times of the control plasmid strain respectively. Therefore, these three proteins Ptc4, Zwf1 and Sme1 and Sod1 were selected for further analysis.

### Overexpressed Ptc4, Sme1 and Sod1 are stable in yeast after prolonged chronological ageing

**Figure 2:**
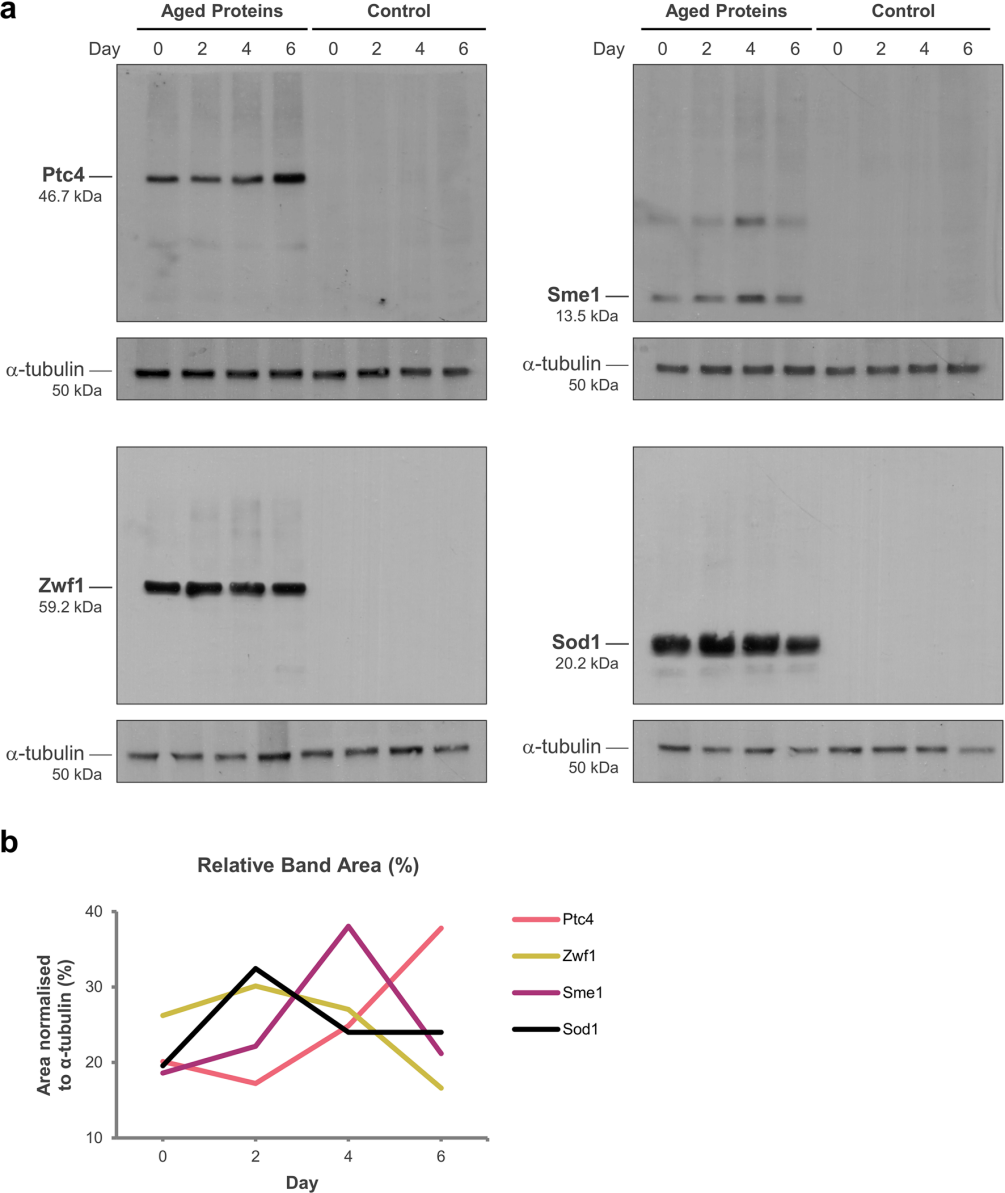
Stabilities of overexpressed proteins Ptc4, Zwf1, Sme1 and Sod1 in yeast during chronological ageing. (**a**) Protein overexpression strains Ptc4, Zwf1, Sme1, Sod1 and control plasmid pYEX strain were chronologically aged. The cultures were harvested and standardised to A_600_ 0.4. The standardised cultures were lysed for sodium dodecyl sulphate–polyacrylamide gel electrophoresis **(**SDS-PAGE) and western blot detection on day 0, 2, 4, and 6. Western blotting was performed using anti-His-tag and HRP-conjugated antibodies. The housekeeping protein α-tubulin were detected on the same membranes using anti-α-tubulin and HRP-conjugated antibodies. The full-length blots are included in Supplementary Fig. S4. (**b**) The protein band intensities representing amount of expressed proteins were quantified by ImageJ software and normalised with internal loading control α-tubulins. The band intensities were expressed as area percentages after normalisation.

To determine the stabilities of the overexpressed proteins (Ptc4, Zwf1, Sme1, Sod1) in yeast during chronological ageing, we performed western blot analysis on the aged cultures. The yeast cells were harvested and standardised at each indicated time-point (day 0, 2, 4, and 6) and lysed to release proteins for western blot detection using antibodies specific to 6 × His-tag on the proteins. The protein band intensities representing expressed protein amounts were quantified and expressed as area percentages using ImageJ software. By comparing the area percentages of the bands on the blots between day 0 and day 6, we observed that Ptc4, Sme1 and Sod1 were stable after prolonged ageing as indicated by increased area percentage by 17.7%, 2.6% and 4.4% respectively on day 6 compared to day 0 while Zwf1 was instable after prolonged ageing as indicated by reduced area percentage by 9.6% on day 6 compared to day 0 (Fig. 2).

### Ptc4 contributes to stress tolerance after prolonged chronological ageing upon protein overexpression

Certain proteins are known to regulate longevity and stress response during chronological ageing^9,10,39^. To examine the stress response of yeast cells with overexpressed Ptc4, Zwf1, Sme1 or Sod1 after prolonged ageing in stressed conditions, thermal and oxidative stress assays were conducted. On day 6 of stationary phase, the aged yeast cells of all the samples were standardised. In the treatment of thermal stress, the standardised aged yeast cells were heated at 55 °C while in the treatment of oxidative stress, the standardised aged yeast cells were treated with 2 mM H_2_O_2_. The nonstress- and stress-treated cells were spread on SC-Ura-Leu agar with 500 μM of CuSO_4_ in ten-fold serial dilution. After stress exposure, the agar plates were then incubated for colony counting.

**Figure 3:**
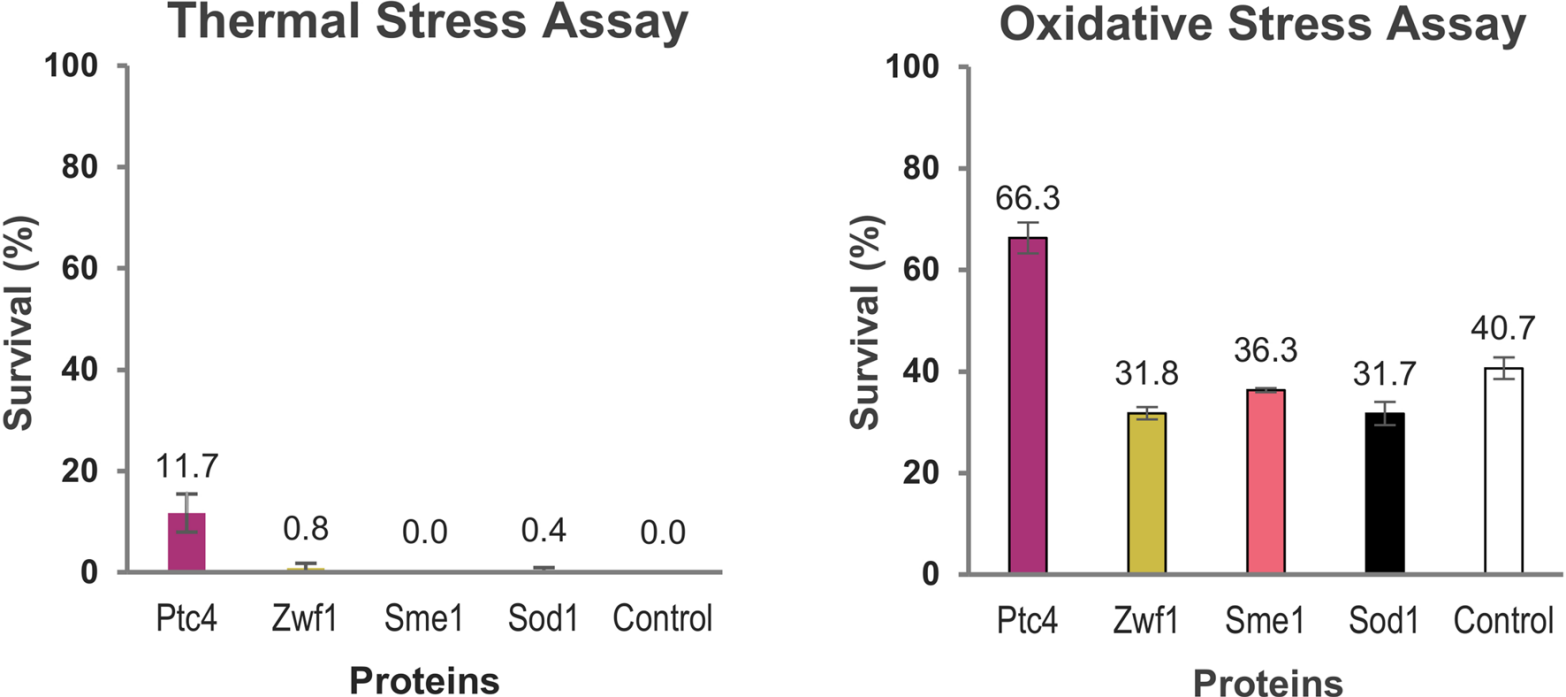
CFU assay of thermal and oxidative stress responses of overexpression strains Ptc4, Zwf1, Sme1 and Sod1 after prolonged ageing. Protein overexpression strains Ptc4, Zwf1, Sme1, Sod1 and strain carrying control plasmid pYEX were chronologically aged up to day 6. The strains were standardised to A_600_ 0.4 and exposed to thermal stress at 55 °C or exposed to oxidative stress by pre-treatment with 2 mM H_2_O_2_. The nonstress- and stress-treated strains were spread in ten-fold serial dilutions (10^–2^, 10^–3^, 10^–4^, 10^–5^) on SC-Ura-Leu agar with 500 μM of CuSO_4_ (2 mM H_2_O_2_ added in agar for H_2_O_2_-treated cells). Colonies formed by the standardised strains at A_600_ 0.4 were counted and converted to survival percentages. Error bars represent the standard deviations of three biological replicates.

We observed that the day 6-aged Ptc4 overexpression strain showed thermal stress tolerance by maintaining 11.7% survival of cells compared to the control plasmid strain with 0% survival and oxidative stress tolerance by maintaining 66.3% survival which was 1.6-times of the control plasmid strain survival (Fig. 3). The other protein expression strains did not show any stress tolerance after prolonged chronological ageing. Taken together these results indicate that Ptc4 is involved in stress response pathway that allows most of the aged cells to survive, whereas Zwf1, Sme1 or Sod1 overexpression does not sustain higher survival after prolonged ageing upon thermal or oxidative stress treatment.

### Ptc4 and Zwf1 promote cell proliferation upon protein overexpression

To further understand the proteins that extended yeast CLS, we examined the effects of their expression levels on the growth of yeast. Each protein overexpression strain and control plasmid strain were grown in SC-Ura-Leu media with different concentration of Cu in a 96-well microplate. Absorbances (A_600_) were measured every 30 min up to 24 h using the Bio Microplate Reader HiTS. The cultures were standardised before measuring absorbance. We observed that when CuSO_4_ concentration was increased (4 μM–500 μM), the slopes of the growth curves shifted to the right (Supplementary Fig. S5). These rightward shifts were expected as Cu is known to have a toxic effect on yeast.

**Figure 4:**
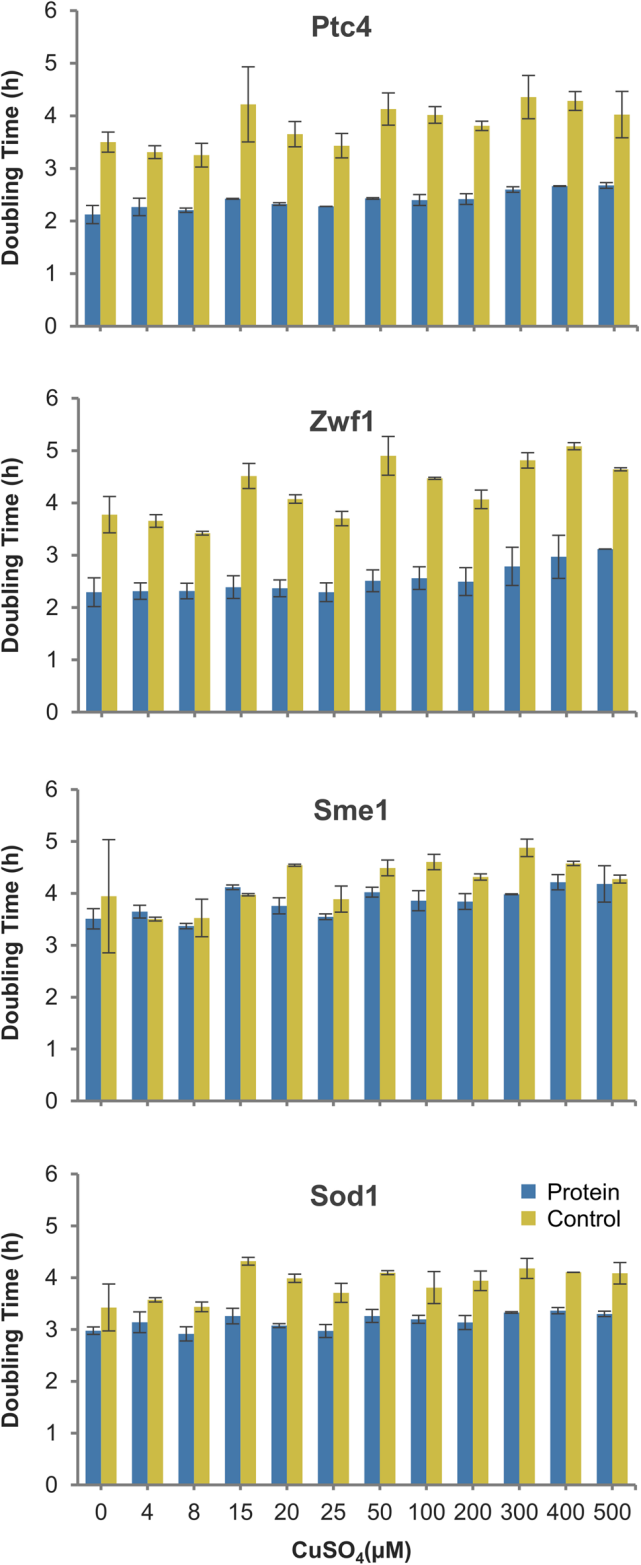
Doubling times of protein overexpression strains Ptc4, Zwf1, Sme1, Sod1 compared to control plasmid pYEX strain under varied inducer (CuSO_4_) concentrations. Protein overexpression strains Ptc4, Zwf1, Sme1, Sod1 and control plasmid strain were grown in SC-Ura-Leu media with different Cu concentration in a 96-well microplate with continuous shaking for 24 h in Bio Microplate Reader HiTS. Absorbances (A_600_) were measured by the reader every 30 min up to 24 h. The bar charts shown here were derived from the growth curves in Supplementary Fig. S5. Error bars represent the standard deviations of two biological replicates.

Initially, we were concerned that the CLS extension might be due to the neutralisation of Cu toxicity by the overexpressed proteins. Therefore, we attenuated protein expression in the strains by varying Cu concentration. The results show that Ptc4 and Zwf1 overexpression strains consistently have shorter doubling times (Fig. 4). In contrast, Sod1 and Sme1 overexpression strains have similar doubling times as the control strain. The rapid proliferation indicated by shorter doubling time of Ptc4 and Zwf1 overexpression strains (Fig. 4) even when no Cu was added indicates that basal expression occurred, and low concentrations of both proteins were enough to enhance cell proliferation. Overall, these results show that overexpression of Ptc4 or Zwf1 in yeast promotes rapid cell proliferation during cell growth.

Additionally, to ensure that the absorbance value corresponded to cell concentration, we also performed cell count. Overnight yeast cultures grew in SC-Ura-Leu supplemented with 100 μM CuSO_4_ in universal bottles. When the cultures reached A_600_ of ~ 0.2, each culture of cells was counted using a haemacytometer (*MA*RIENFELD). The cell count showed that the number of cells from different samples at A_600_ of ~ 0.2 was in the range of 4–5 × 10^7^ cells/mL (Supplementary Fig. S6). Therefore, absorbance values are related to cell concentrations and not to other factors.

## Discussion

Proteins that can extend yeast CLS had been identified previously using different methods. For example, CLS extension by the overexpression of Sod1 and Sod2 was discovered using CFU count method in a live/dead fluorescent assay^10^ and the Ecl family genes, which extended CLS in fission yeast, were identified using relative colony count against cell turbidity^40–42^. However, there are still other proteins whose roles in CLS remain unknown.

In this study, we began by reviewing the literature for ageing-related proteins whose roles in CLS have not been reported and selected 15 proteins based on their gene ontology, protein function, subcellular location, and residue length for a protein overexpression CLS assay screen. These 15 screened proteins play different roles in cellular functions such as osmotic, oxidative and/or thermal stress adaptation, pheromone response pathway, filamentation pathway, pre-mRNA splicing, pre-mRNA polyadenylation, protein folding, ubiquitin–proteasome system, DNA replication, ATP synthesis, apoptosis and cell cycle progression (Supplementary Table S1). Our CLS screen shows that the overexpression of Ptc4, Zwf1, Sme1, Cpr3, Kss1, Sod1 or Ump1 can extend yeast CLS (Fig. 1c). The CLS extensions by the top three overexpressing proteins Ptc4, Zwf1 and Sme1 and the positive control Sod1 were validated in CFU assay (Fig. 1d). Moreover, *ptc4*∆, *zwf1*∆, *sme1*∆ or *sod1*∆ decreased yeast CLS (Fig. 1e), consistent to previous findings whereby *zwf1*∆^43^ or *sod1*∆^26^ in *S. cerevisiae* decreased yeast CLS. These findings thus support the importance of the genes encoding proteins Ptc4, Zwf1, Sme1 and Sod1 in regulating CLS. Sod1 is known to extend yeast CLS upon overexpression but has an opposite effect on yeast replicative life span (RLS) by causing a reduction in yeast RLS when overexpressed^26^, while the effects of the other proteins Ptc4, Zwf1 and Sme1 on yeast RLS are still unknown. Therefore, it is pertinent to perform RLS assays to determine the effects of the proteins Ptc4, Zwf1 and Sme1 on RLS in yeast. We further determined the intracellular stabilities of the top three CLS-extending proteins Ptc4, Zwf1, Sme1 during chronological ageing by western blotting. Next, we found that Ptc4 contributed to thermal and oxidative stress tolerances. Lastly, we investigated the effects of protein concentrations on rate of cell proliferation and found that Ptc4 and Zwf1 promoted rapid cell proliferation even at basal protein expression level.

Ageing can be caused by a reduction in the ability of cells to overcome environmental stress^44^. High level of reactive oxygen species (ROS) has been suggested as the main contributor of ageing^45,46^. ROS can lead to oxidative damage of biomacromolecules^47,48^. In addition, downregulation of thermal stress response that occurs during ageing may result in the accumulation of damaged proteins^44^. Our result shows that the day 6-aged Ptc4 overexpression strain contributes to thermal and oxidative stress tolerances (Fig. 3). Likewise, we also investigated the thermal and oxidative stress tolerances of the Ptc4 overexpression strain at mid-log phase of cell growth. The Ptc4 overexpression strain at mid-log phase showed thermal stress tolerance relatively by maintaining 8% of survival while the survival of control plasmid strains was 0%. The Ptc4 overexpression strain at mid-log phase demonstrated oxidative stress tolerance at low H_2_O_2_ concentration (100 μM and 500 μM) (Supplementary Fig. S7). We found that the yeast cells at mid-log phase could not withstand thermal stress at 55 °C for 30 min and above. In the case of oxidative stress, most of the yeast cells at mid-log phase could not survive after being exposed to 1 mM H_2_O_2_, which were unlike the chronologically aged yeast cells which could sustain survival at higher H_2_O_2_ concentration of 2 mM. These observations were consistent to the idea that elevated stress resistance occurs upon cell entry into stationary phase for long-term cell survival^25^.

The loss of Ptc4 in *Schizosaccharomyces pombe* caused cell death upon H_2_O_2_ stress exposure^49^, indicating the importance of Ptc4 in the regulation of oxidative stress response. The stress tolerances contributed by Ptc4 in thermal and oxidative stress assays indicate its possible role in regulatory pathway mediating thermal and oxidative stress responses and this role may directly contribute to higher CLS extension. The proteins, Zwf1 and Sod1 are known to confer oxidative stress tolerance in *S. cerevisiae*^50–52^. Therefore, we expected that Zwf1 or Sod1 overexpression yeast strain can maintain higher survival in oxidative stress condition after prolonged ageing. Surprisingly, the oxidative stress tolerance of the day 6-aged Zwf1 or Sod1 overexpression strain was downregulated, and no thermal stress tolerance was observed in these strains (Fig. 3). The decreased oxidative stress tolerance of Zwf1 overexpression strain might be due to Zwf1 degradation after prolonged ageing (Fig. 2b). Likewise, the Sme1 overexpression strain has the same stress response as the Zwf1 or Sod1 overexpression strain after prolonged ageing. The stress sensitivity of the day 6-aged Zwf1, Sme1 or Sod1 overexpression strain might explain their lower survival on day 6 compared to Ptc4 overexpression strain (Fig. 1d). As such, stress tolerance can be reduced or lost after prolonged ageing and shows impact on cell survival.

Copper is a well-studied important element required by all living organisms, but Cu accumulation is highly toxic^53^. Cu toxicity can be attributed to its involvement in Fenton-like reactions that produce hydroxyl radicals. Hydroxyl radicals cause cellular damage such as DNA and RNA cleavage, protein oxidation, and membrane damage due to lipid peroxidation^54,55^. In our assays, Cu was added into the growth media to induce protein expression. Initially, we were concerned that CLS extension by Ptc4, Zwf1, Sme1 and Sod1 was due to the neutralisation of Cu toxicity by the expressed proteins. However, we noticed that the growth of the protein overexpression strains and control plasmid pYEX strain became slower at increasing concentration of Cu (Supplementary Fig. S5), most likely due to Cu toxicity. The slower growth of all strains at increasing Cu concentration implies that CLS extension was not due to neutralisation of Cu toxicity, but by other means.

Our data reveal that yeasts with overexpressed Ptc4 or Zwf1 can promote cell proliferation despite the higher Cu concentration. Hence, it is likely that Cu does not diminish the function of overexpressed Ptc4 and Zwf1 to induce rapid cell proliferation even though cell growth is affected. We also observed that the yeast strain carrying recombinant plasmids with *PTC4* or *ZWF1* demonstrated rapid cell proliferation when growing in the medium without the addition of Cu (Fig. 4). We suspect that basal expression occurred due to the induction by trace amounts of Cu readily available in SC media and the expression level can be enhanced by the high copy number of the plasmid induced by leu2-d^56^. Therefore, we investigated the expression level of representative Ptc4 induced at different concentration of Cu. As expected, Ptc4 without addition of Cu was detected and its expression level was increased at increasing concentration of Cu (Supplementary Fig. S8). The increase in cell proliferation rate by the basal expression of Ptc4 or Zwf1 is noteworthy for two reasons—(i) the lower protein concentrations are likely closer to natural cellular levels, and (ii) that the proteins can induce a positive effect on rate of cell proliferation at minimal concentration.

Hereby, we make a presumed correlation between the roles of the proteins Ptc4, Zwf1, Sme1 and Sod1 and their effects on rate of cell proliferation and CLS. Ptc4 plays a role as a negative regulator of the high-osmolarity glycerol (HOG) pathway, thus it can inhibit the negative regulation of cell-wall construction which is essential for cell growth and proliferation, caused by cellular osmotic or oxidative stress-induced HOG pathway^57^. This negative regulator role of Ptc4 may contribute to rapid cell proliferation. Moreover, deletion of *HOG1* in *S. cerevisiae* extends CLS and confers thermal and oxidative stress tolerances^49,58^, suggesting that inactivation of Hog1 through dephosphorylation by Ptc4 contributes to CLS extension and stress tolerance for long-term cell survival. In the case of Zwf1, its overexpression may enhance the pentose phosphate pathway to generate reduced nicotinamide adenine dinucleotide phosphate (NADPH)^59,60^. NADPH acts as an electron donor to maintain cellular redox homeostasis through thioredoxin and glutathione reductase systems. The thioredoxin and glutathione reductase systems can decrease oxidative damage to thiol-containing proteins in yeast cells. Thus, the reduced oxidative damage may allow the cells to grow healthier, proliferate faster and extend CLS^61–63^. Overexpression of Sod1 had been reported to promote minor CLS extension in budding yeast^10^. In consistent, our screen shows Sod1 can extend yeast CLS (Fig. 1c, 1d). Sod1 plays the same role as Zwf1 to allow cellular adaptation in oxidative stress. Sod1 catalyses the dismutation of superoxide anions to hydrogen peroxide and oxygen^52,64^, subsequently protecting mitochondria against oxidative damage^65^ and extending CLS. Sme1 is one of the Sm proteins that bind to small nuclear RNAs (snRNAs) to form RNA–protein complexes, the small nuclear ribonucleoproteins (snRNPs) in spliceosomes^66,67^. Pre-mRNA splicing is executed by the spliceosomes through removal of introns and combination of exons in pre-mRNA and is essential for preparation of mature mRNA for translation. Several studies had reported that splicing regulation and changes in splicing factor expression are associated to cellular senescence^68^ and ageing in mice and humans^69–71^. Thereby, overexpression of Sme1 may enhance proper splicing activity for producing proteins that enable cells to counteract the damage increased with ageing, thus promoting CLS extension in yeast.

In this screen of ageing-related proteins, we identified several proteins that extended yeast CLS. Notably, we found that cell proliferation rate was increased by minimal concentration of Ptc4 and Zwf1, and that the proteins act on different pathways to promote rapid cell proliferation. Future studies should focus on the identification of effector mechanisms that show how these proteins protect yeast against chronological ageing. Furthermore, whether the induction of rapid cell proliferation is beneficial for yeast to delay senescence is also worthy of its own investigation.

## Supplementary Information

Supplementary Information.

## Data Availability

The datasets generated during and/or analysed during the current study are available from the corresponding author on reasonable request.
