# Supplementary material for: Screening of selected ageing-related proteins that extend chronological life span in yeast Saccharomyces cerevisiae
Source: Sci Rep. 2021 Dec 17;11:24148. doi: 10.1038/s41598-021-03490-7 (PMC8683414; doi:10.1038/s41598-021-03490-7)
Supplement: Supplementary file 1 — Supplementary Information. [file 41598_2021_3490_MOESM1_ESM.pdf]

Screening of selected ageing-related proteins that extend chronological life span in yeast *Saccharomyces cerevisiae*

Jee Whu Lee[1,2], Tee Gee Ong[1,2], Mohammed Razip Samian[2,3], Aik-Hong Teh[2,4], Nobumoto Watanabe[2,5], Hiroyuki Osada[2,6] and Eugene Boon Beng Ong[1,2*]

[1]Institute for Research in Molecular Medicine (INFORMM), Universiti Sains Malaysia.

[2] USM-RIKEN International Centre for Ageing Science (URICAS).

[3]School of Biological Sciences, Universiti Sains Malaysia.

[4]Centre for Chemical Biology, Universiti Sains Malaysia.

[5]Bioprobe Application Research Unit, RIKEN Centre for Sustainable Resource Science, RIKEN, Japan.

[6]Chemical Biology Research Group, RIKEN Centre for Sustainable Resource Science, RIKEN, Japan.

*Corresponding author at: Institute for Research in Molecular Medicine (INFORMM), Universiti Sains Malaysia.

email: eugene@usm.my (E. Boon Beng Ong).

Supplementary Materials

Supplementary Table S1. Ageing-related genes encoded proteins selected for overexpression in the chronological life span assay.

Gene	Systematic name	Location	Main function	Human homolog
PTC4	YBR125C	Cytoplasm	Encodes type 2C Ser/Thr phosphatase (Ptc4) which dephosphorylates high osmolarity-induced Hog1 in high osmolarity glycerol (HOG) pathway	Protein phosphatase, Mg^{2+}/Mn^{2+} dependent 1G (PPM1G)
ZWF1	YNL241C	Cytoplasm	Encodes glucose-6-phosphate dehydrogenase (G6PD/Zwf1) enzyme of pentose phosphate pathway (PPP), which produces nicotinamide adenine dinucleotide phosphate (NADPH) required for oxidative stress adaptation by detoxifying reactive oxygen species (ROS) and methionine biosynthesis	Glucose-6-phosphate dehydrogenase (G6PD)
SME1	YOR159C	Nucleus	Encodes Sm protein E which is one of the Sm core complex proteins (B, B′, D1, D2, D3, E, F, G). The Sm proteins bind to small nuclear RNAs (snRNAs) to produce small nuclear ribonucleoprotein (snRNPs). SnRNP-E is essential for U1, U2, U4 and U5 snRNA stability, cap modification and pre-messenger RNA (mRNA) splicing for protein synthesis	Human E core protein
CPR3	YML078W	Mitochondrion	Encodes yeast mitochondrial cyclophilin (Cpr3) which is essential for protein folding via catalysing peptidylprolyl cis-trans isomerization and lactate metabolism at high temperature	CypD
KSS1	YGR040W	Nucleus	Encodes mitogen-activated protein kinase (Kss1) which regulates activation/repression	Erk2

			of pheromone response pathway and nutrient starvation induced-filamentation pathway	
POL30	YBR088C	Nucleus	Encodes proliferating cell nuclear antigen (PCNA/Pol30) which is involved in DNA replication, DNA damage repair such as nucleotide excision repair (NER), postreplicational DNA repair, base excision repair and mismatch repair (NMR) and cell cycle control	PCNA
SOD1	YJR104C	Nucleus, cytosol, mitochondrial intermembrane space	Encodes Cu/Zn superoxide dismutase (Sod1) that catalyzes the dismutation of superoxide anions to hydrogen peroxide and oxygen to protect mitochondria from oxidative damage	Sod1
AIM14 or *YNO1*	YGL160W	Endoplasmic reticulum	Encodes yeast NADPH oxidase 1 (Aim14/Yno1) that catalyzes the generation of superoxide from NADPH and oxygen in endoplasmic reticulum and regulates actin cytoskeleton. Overexpression of Aim14 produces reactive oxygen species (ROS) that results in apoptosis or programmed cell death (PCD)	-
RPN11	YFR004W	Cytosol, mitochondrion, nucleus, proteasome storage granule	Encodes subunit of 19S regulatory particle (RP), Rpn11, which is assembled with 20 S proteolytic core particle (CP) in 26S proteasome. Rpn11 is a proteasome deubiquitinase that catalyzes the detachment of ubiquitin chains from proteasome substrate proteins to promote ubiquitinated protein degradation. Overexpression of Rpn11 regains 26S proteasome	PSMD14

			activity, leading to life span extension of flies	
PAP1	YKR002W	Nucleus	Encodes poly(A) polymerase, Pap1, that is involved in generation of poly(A) tail at 3' end of messenger RNA precursors (pre-mRNAs). Poly(A) tail is essential for facilitating the exit of mature mRNAs from nucleus to cytoplasm, enhancing translation of mRNAs and protecting mRNAs from degradation	PAPOLA, PAPOLB, PAPOLG
HSC82	YMR186W	Cytoplasm, mitochondrion, plasma membrane	A paralog of *HSP82*, which encodes heat shock protein 90 (Hsp90) that enhances hyperthermic-induced rDNA hypercondensation and enables cell cycle progression at elevated temperatures. Additionally, Hsp90 is essential for vesicular transport at normal growth temperature, cell division progression at increased temperature, telomere length maintenance and is involved in negative regulation of heat shock response	Hsp90α or Hsp90β
UBP13	YBL067C	Cytoplasm, nucleus	Encodes ubiquitin C-terminal hydrolase, Ubp13, that is essential for respiration, biosynthesis of mitochondrial ATP synthase subunit 9 (Atp9) which is important for ATP synthase to generate ATP from ADP and phosphate and confers cold tolerance in *Saccharomyces cerevisiae*	Usp12
CDC6	YJL194W	Cytoplasm, nucleus	Encodes ATP/GTPase, Cdc6, controlling S phase initiation that is essential for initiating DNA replication. Cdc6 ATP hydrolysis activity is required for yeast cell growth or	Cdc6

			survival and also for disengagement of Cdc6 from pre-replicative complex [origin recognition complex (ORC), Cdc6, and Cdt1] to induce DNA replication	
UMP1	YBR173C	Nucleus (around nuclear envelope)	Encodes 20S proteasome maturase, Ump1, which is essential for biogenesis of the 20S proteasome, a catalytic core of the 26S proteasome in ubiquitin-proteasome system. Ump1 maintains mitochondrial genome stability. Ump1 reduces protein oxidation, promotes proteasome-mediated protein degradation, and extends chronological life span. Additionally, Ump1 is inducible by DNA damage and confers ultraviolet light tolerance to required *S. cerevisiae* cells.	POMP
ESP1	YGR098C	Nucleus	Encodes caspase-like protease, Esp1 (separase/separin), that is essential to cleave cohesin subunit, Mcd1 (Scc1) and thus allow sister chromatid separation and initiate anaphase onset. Cleavage of Mcd1 by Esp1 also triggers hydrogen peroxide-induced apoptosis in budding yeast. Additionally, Esp1 promotes anaphase spindle elongation and release of Cdc14 phosphatase from nucleolus to enable mitotic exit.	ESPL1

Supplementary Table S2. DNA sequences of cloning plasmid pYEX and control plasmid pYEX.

Plasmid	Sequence
Cloning pYEX	*Sal*I Homologous Region I *Pst*I *Sac*II *Xho*I *Nhe*I *Sac*I *Xma*I<br>5'-GTCGACGGTGGTTCTGGTGGCGGCTCTGGCCTGCAGCCGCGGCTCGAGGCTAGCGAGCTCCCCGGGCCTC<br>Homologous Region II FLAG-tag *Bam*HI 6x His-tag<br>CTCCATACCAGCCTCTCGGAGGAGGAGGAAGCGATTACAAAGACGATGACGACAAGGGATCCCATCATCA<br>Stop<br>CCATCACCATTGA-3'
Control pYEX	*Sal*I Homologous Region I *Sac*II Start Homologous Region II<br>5'-GTCGACGGTGGTTCTGGTGGCGGCTCTGGCCCGCGGATGCCTCCTCCATACCAGCCTCTCGGAGGAGGAGG<br>FLAG-tag *Bam*HI 6x His-tag Stop<br>AAGCGATTACAAAGACGATGACGACAAGGGATCCCATCATCACCATCACCATTGA-3'

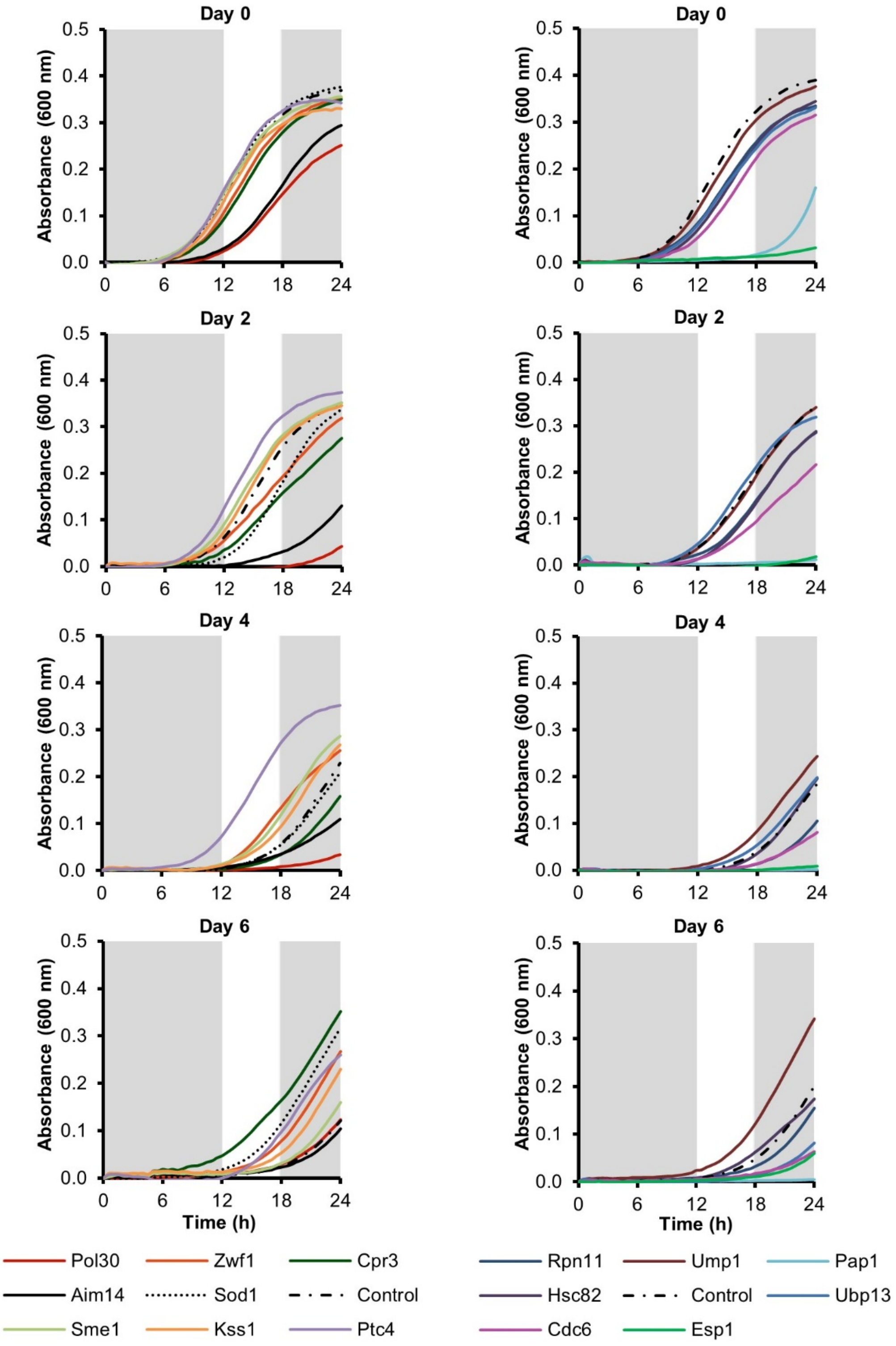

Supplementary Fig. S1. Primary screen of 15 proteins overexpressed in yeast. Outgrowth curves in primary screen were developed from absorbance measurement of yeast strain MLC30M with overexpressed protein (Pol30, Zwf1, Cpr3, Ptc4, Sme1, Kss1, Rpn11, Pap1, Hsc82, Ubp13, Cdc6, Esp1, Sod1, Aim14 or Ump1) or control plasmid pYEX using Bio Microplate Reader HiTS. Absorbance (A_{600}) was measured every 30 min up to 24 h from day 0 until day 6. The absorbance value of each protein overexpression strain and control plasmid strain was average value of three biological replicates.

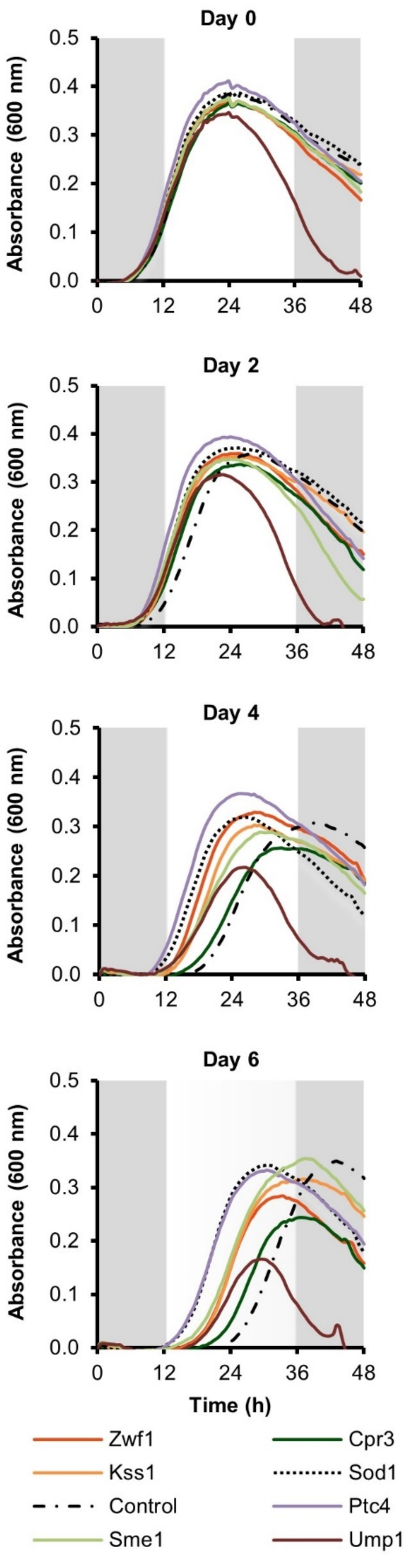

Supplementary Fig. S2. Confirmatory screen of seven proteins overexpressed in yeast. Outgrowth curves in confirmatory screen were developed from absorbance measurement of yeast strain MLC30M with overexpressed protein (Ptc4, Zwf1, Sme1, Cpr3, Kss1, Sod1 or Ump1) or control plasmid pYEX using Bio Microplate Reader HiTS. Absorbance (A_{600}) was measured every 30 min up to 48 h from day 0 until day 6. The absorbance value of each protein overexpression strain and control plasmid strain was average value of three biological replicates.

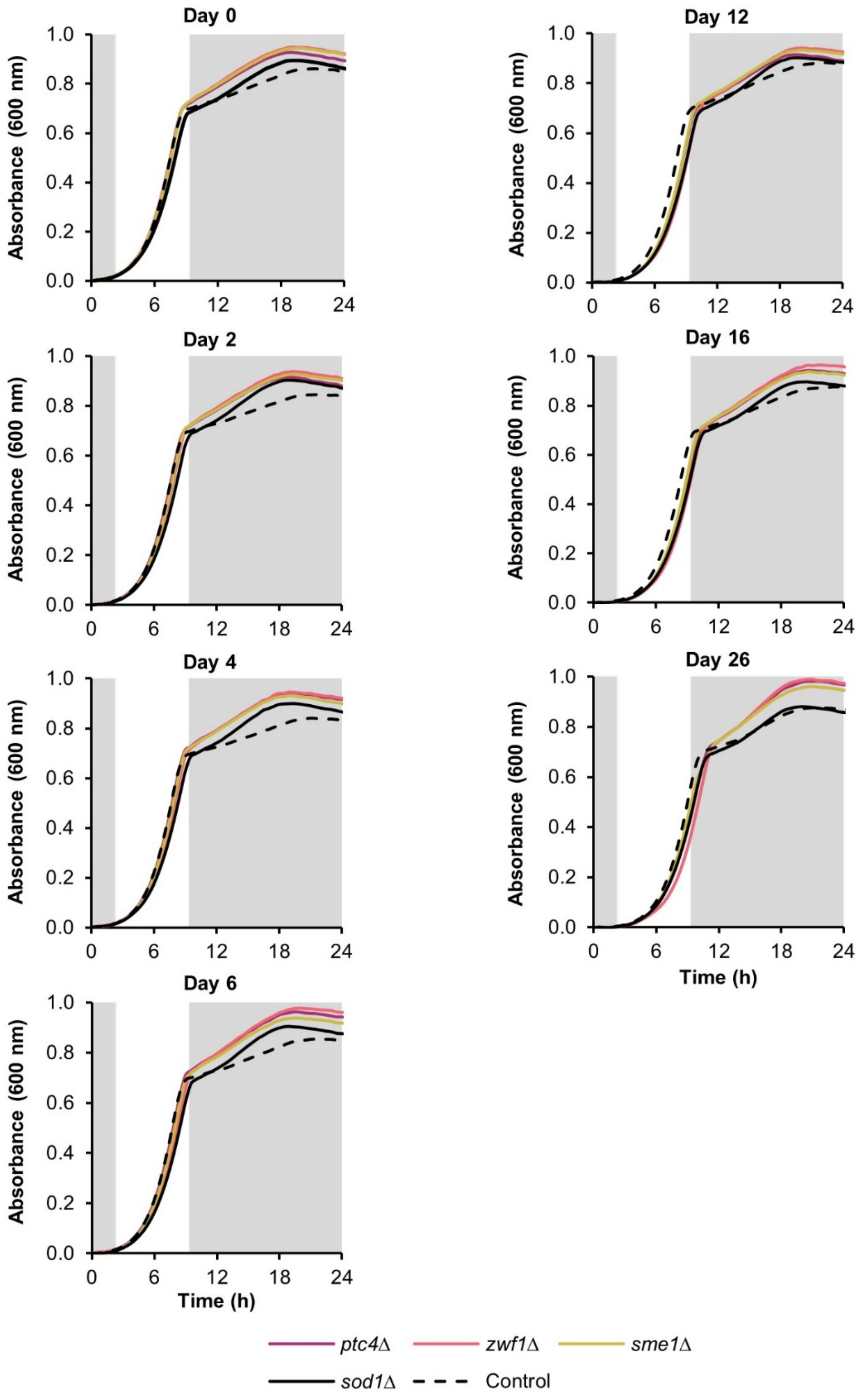

Supplementary Fig. S3. CLS screen of gene deletion mutants. Outgrowth curves were developed from absorbance measurement of *ptc4*Δ, *zwf1*Δ, *sme1*Δ, *sod1*Δ mutants and wild-type yeast strain BY4741 using Bio Microplate Reader HiTS. Absorbance (A_{600}) was measured every 30 min up to 24 h from day 0 until day 26. The absorbance value of each strain was average value of three biological replicates.

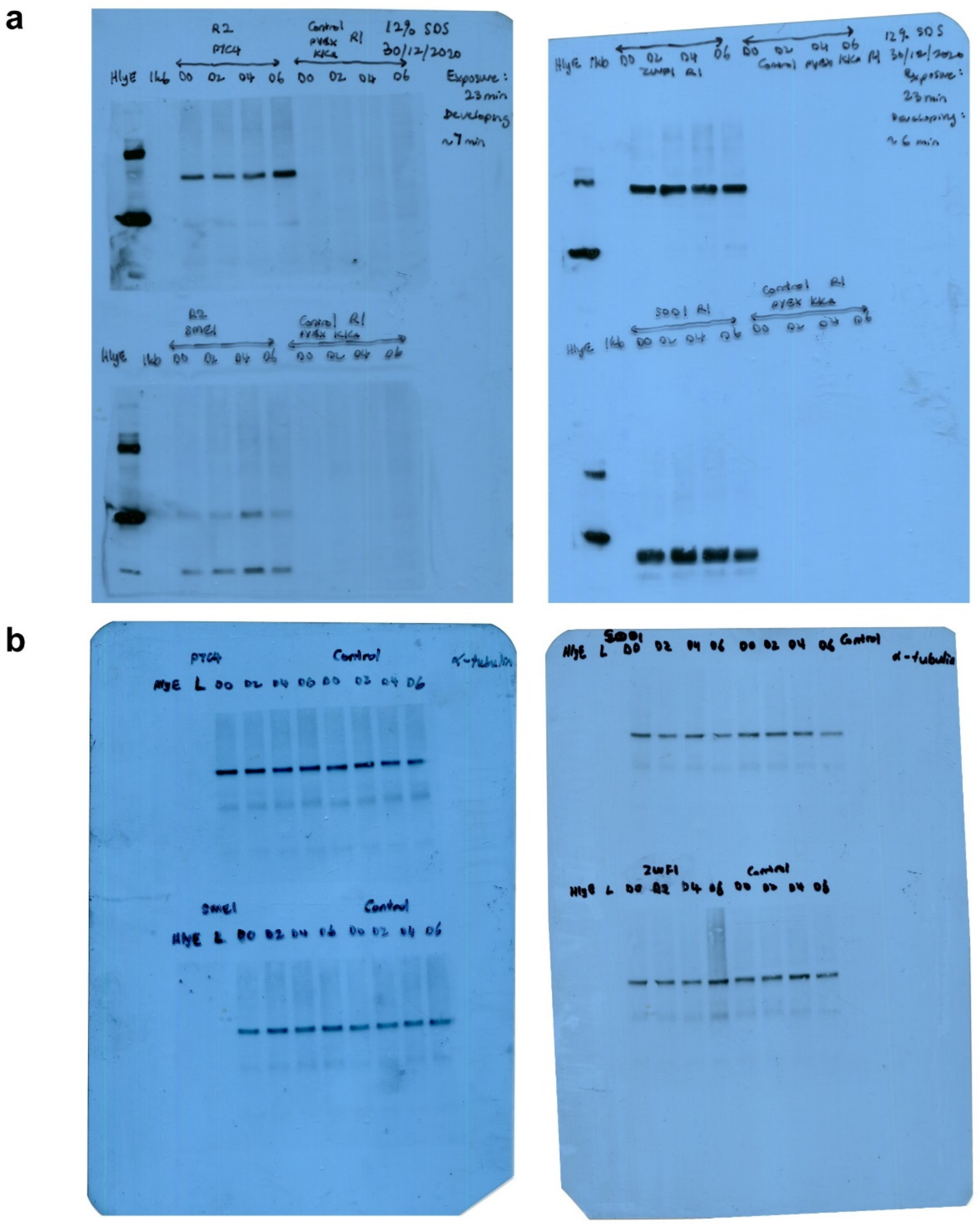

Supplementary Fig. S4. Stabilities of overexpressed proteins Ptc4, Zwf1, Sme1 and Sod1 in yeast during chronological ageing. Protein overexpression strains Ptc4, Zwf1, Sme1, Sod1 and control plasmid pYEX were chronologically aged. An amount of 750 µL culture was harvested and standardised to A_{600} 0.4. The standardised culture each was lysed and the intracellular proteins in supernatant were separated via SDS-PAGE. Chromatein prestained protein ladder (Vivantis, Malaysia) was used. The proteins on SDS gels were transblotted onto nitrocellulose membranes. (a) The day 0, 2, 4, and 6 proteins on nitrocellulose membranes were detected using anti-His-tag and horseradish peroxidase (HRP)-conjugated antibodies. Purified Haemolysin E (HlyE) with C-terminal 6× His-tag at 34.0 kDa was a positive control for anti-His-tag antibody detection while a short peptide with C-terminal 6× His-tag expressed from control (C) plasmid pYEX at 3.2 kDa was a negative control for western blot detection. (b) The loading control α-tubulins at ~50.0 kDa on the same membranes were detected using anti-α-tubulin and HRP-conjugated antibodies.

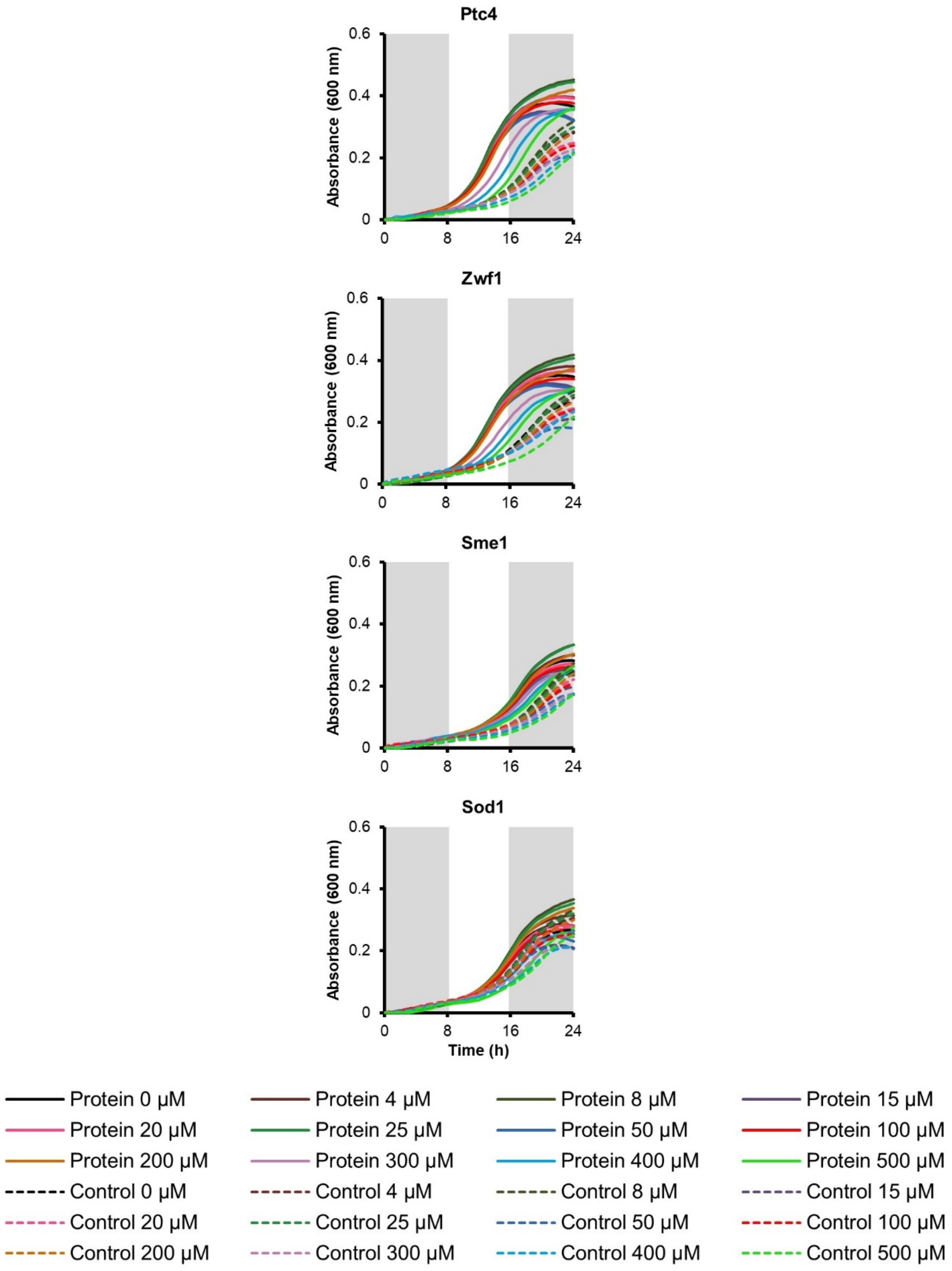

Supplementary Fig. S5. Growth curves of yeast strains at increasing concentration of $CuSO_4$. Growth curves were developed from absorbance measurement of yeast strain MLC30M with overexpressed protein (Ptc4, Zwf1, Sme1 or Sod1) or control plasmid pYEX using Bio Microplate Reader HiTS. The cultures were incubated in SC-Ura-Leu with different concentration of $CuSO_4$ in a 96-well microplate. Absorbance (A_{600}) was measured every 30 min up to 24 h during incubation. The absorbance value of each protein overexpression strain and control plasmid strain was average value of two biological replicates.

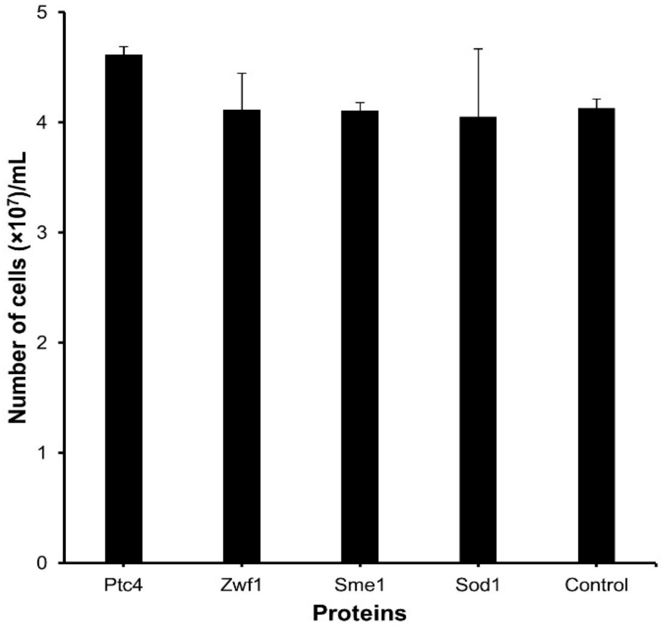

Supplementary Fig. S6. Cell concentration at A_{600} of ~0.2. Overnight yeast cultures grew in universal bottles containing SC-Ura-Leu supplemented with 100 µM $CuSO_4$ until reaching A_{600} of ~0.2. An amount of 10 µL of each protein (Ptc4, Zwf1, Sme1 or Sod1) overexpression strain and control plasmid strain at A_{600} of ~0.2 was counted using a haemacytometer. Error bars represent the standard deviations of two biological replicates. Averaged duplicate countings were taken for each replicate.

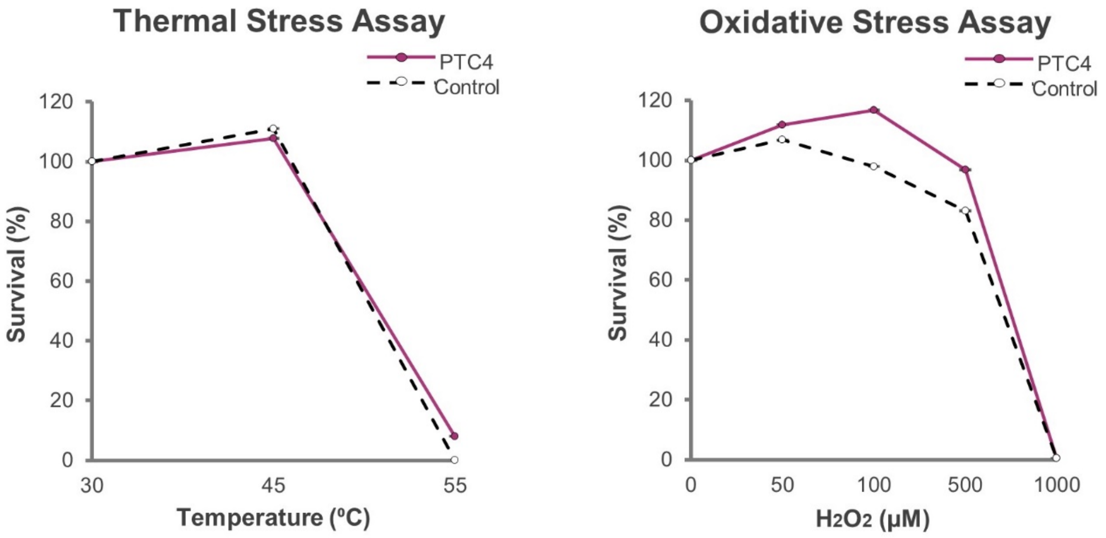

Supplementary Fig. S7. CFU assay for Ptc4 overexpression strain and control plasmid pYEX strain at mid-log phase under treatment of thermal or oxidative stress. Ptc4 overexpression strain and control plasmid strain were grown in SC-Ura-Leu medium with 8 µM $CuSO_4$ to mid-log phase at A_{600} 0.2-0.25. The strains were exposed to thermal stress for 15 min at 45°C or 55°C, or exposed to oxidative stress by being pretreated with H_2O_2 (50, 100, 500, 1,000 µM) for 15 min at 30°C. The nonstress- and stress-treated strains in ten-fold serial dilutions (10^{-2}, 10^{-3}, 10^{-4}, 10^{-5}) were spread on SC-Ura-Leu agar with 8 µM of $CuSO_4$ (H_2O_2 at respective concentration added into agar for H_2O_2-treated cells) and incubated at 30°C. Colonies formed were counted. The survivals of nonstress-treated strains were expressed as 100 %. Error bars represent the standard deviations of three biological replicates.

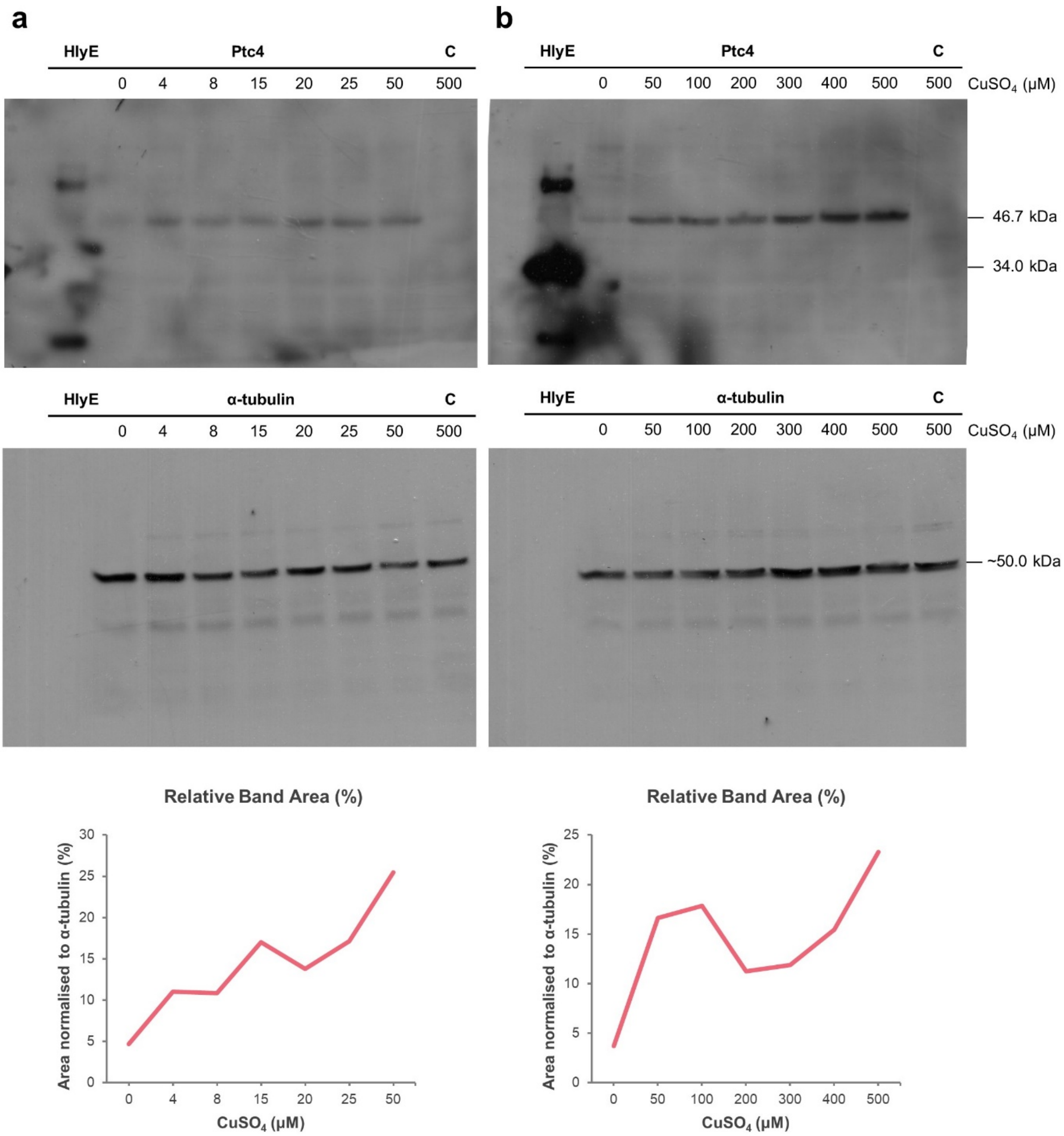

Supplementary Fig. S8. Ptc4 expression level in a range of $CuSO_4$ concentration. Strains with overexpressed Ptc4 at 46.7 kDa grew in universal bottles containing SC-Ura-Leu supplemented with (a) 0, 4, 8, 15, 20, 25, 50 μM of $CuSO_4$ or (b) 0, 50, 100, 200, 300, 400, 500 μM of $CuSO_4$ while control (C) plasmid pYEX strain grew in universal bottle containing SC-Ura-Leu supplemented with 500 μM of $CuSO_4$ until reaching A_{600} of ~0.2. An amount of 1,400 μL of culture was harvested and standardised to A_{600} 0.4. The standardised culture each was lysed for protein detection through SDS-PAGE and western blotting. Chromatein prestained protein ladder (Vivantis, Malaysia) was used. The proteins on SDS gels were transblotted onto nitrocellulose membranes. The Ptc4 proteins on nitrocellulose membranes were detected using anti-His-tag and HRP-conjugated antibodies. Purified Haemolysin E (HlyE) with C-terminal 6× His-tag at 34.0 kDa was a positive control for anti-His-tag antibody detection while a short peptide with C-terminal 6× His-tag expressed from control (C) plasmid pYEX at 3.2 kDa was a negative control for western blot detection. The loading control α-tubulins on the same

membranes were detected using anti-α-tubulin and HRP-conjugated antibodies. The protein band intensities representing intracellular protein amounts were quantified by ImageJ software and normalised with loading control α-tubulin at ~50.0 kDa. The band intensities were expressed as area percentages after normalisation.
